# The Burden of Bacterial Vaginosis: Women’s Experience of the Physical, Emotional, Sexual and Social Impact of Living with Recurrent Bacterial Vaginosis

**DOI:** 10.1371/journal.pone.0074378

**Published:** 2013-09-11

**Authors:** Jade E. Bilardi, Sandra Walker, Meredith Temple-Smith, Ruth McNair, Julie Mooney-Somers, Clare Bellhouse, Christopher K. Fairley, Marcus Y. Chen, Catriona Bradshaw

**Affiliations:** 1 Department of Epidemiology and Preventative Medicine, Monash University, Melbourne, Australia; 2 Melbourne School of Population and Global Health, The University of Melbourne, Melbourne, Australia; 3 General Practice and Primary Health Care Academic Centre, The University of Melbourne, Melbourne, Australia; 4 Centre for Values, Ethics and the Law in Medicine and Sydney School of Public Health, The University of Sydney, Sydney, Australia; 5 Melbourne Sexual Health Centre, Alfred Health, Melbourne, Australia; Columbia University, United States of America

## Abstract

**Background:**

Bacterial vaginosis is a common vaginal infection, causing an abnormal vaginal discharge and/or odour in up to 50% of sufferers. Recurrence is common following recommended treatment. There are limited data on women’s experience of bacterial vaginosis, and the impact on their self-esteem, sexual relationships and quality of life. The aim of this study was to explore the experiences and impact of recurrent bacterial vaginosis on women.

**Methods:**

A social constructionist approach was chosen as the framework for the study. Thirty five women with male and/or female partners participated in semi-structured interviews face-to-face or by telephone about their experience of recurrent bacterial vaginosis.

**Results:**

Recurrent bacterial vaginosis impacted on women to varying degrees, with some women reporting it had little impact on their lives but most reporting it had a moderate to severe impact. The degree to which it impacted on women physically, emotionally, sexually and socially often depended on the frequency of episodes and severity of symptoms. Women commonly reported that symptoms of bacterial vaginosis made them feel embarrassed, ashamed, ‘dirty’ and very concerned others may detect their malodour and abnormal discharge. The biggest impact of recurrent bacterial vaginosis was on women’s self-esteem and sex lives, with women regularly avoiding sexual activity, in particular oral sex, as they were too embarrassed and self-conscious of their symptoms to engage in these activities. Women often felt confused about why they were experiencing recurrent bacterial vaginosis and frustrated at their lack of control over recurrence.

**Conclusion:**

Women’s experience of recurrent bacterial vaginosis varied broadly and significantly in this study. Some women reported little impact on their lives but most reported a moderate to severe impact, mainly on their self-esteem and sex life. Further support and acknowledgement of these impacts are required when managing women with recurrent bacterial vaginosis.

## Introduction

Bacterial vaginosis (BV) is a common condition affecting between 10%–30% of women in developed nations [1], [2], including the United Kingdom and United States but in excess of 50% of women in rural sub Saharan Africa [3]. Symptoms of BV include vaginal malodour often likened to a ‘fishy odour’ and a thin, off white homogenous vaginal discharge [4], [5]. BV has been associated with serious sequelae including miscarriage, preterm delivery and increased risk of HIV and sexually transmitted infections (STIs) [6]–[8].

While the aetiology of BV is still unclear, it appears to be a polymicrobial condition which is associated with a profound disturbance of the normal vaginal flora. Whether BV is sexually transmitted remains unclear, however epidemiological studies have shown strong evidence of an association between BV and sexual activity [2], [9], [10]. Cross-sectional studies of women who have sex with women (WSW) have also reported a higher prevalence of BV than found in heterosexual women [11]–[15]. Current recommended treatment for BV is with oral or vaginal antibiotics, however current studies have shown recurrence rates of up to 60% within 12 months of treatment [16].

To date, only one other qualitative study has specifically explored the experiences and impact of recurrent BV on women. In a mixed methods study by Payne et al [17] of 23 African American women experiencing recurrent BV, women commonly reported feeling embarrassed and frustrated by the symptoms of BV. Their concerns and self-consciousness around malodour led them to try various self-help remedies, including douching and manual vaginal washing, in order to have a sense of control over their symptoms. Past studies have found that practices such as douching, which are commonly used by women experiencing vaginal conditions, are associated with a higher incidence of bacterial vaginitis [18]–[20]. Overall, the study found that recurrent BV impacted negatively on women’s social, personal and work relationships, significantly affecting their quality of life [17]. These findings are also supported in a broader qualitative study of women’s experiences of vaginitis (thrush, BV and trichomoniasis) in a primary care setting, which also found that vaginal symptoms could cause extreme anxiety and distress to women, impacting heavily on their social and sexual lives [21].

While there is little data on the psychosocial impact of recurrent BV on women, there is considerable data on the adverse psychosocial sequelae experienced by individuals diagnosed with genital herpes and human papillomavirus (HPV). A number of qualitative and mixed methods studies [22]–[24] have found that individuals with genital herpes or HPV commonly experience significant anxiety, self-blame and embarrassment around their diagnosis, and fear disclosing their status to others. Individuals often report feeling stigmatised, experiencing lowered self-esteem and concern around future sexual interaction and relationships [22]–[24].

The aim of this study was to use qualitative research methods to explore the experiences and impact of recurrent BV on women.

## Methods

This study has been reported in accordance to the Consolidated criteria for reporting qualitative research (COREQ) guidelines [25]. Further findings from this study will be reported in an upcoming paper.

### Ethics Statement

Ethical approval for this study was granted by the Alfred Hospital Ethics Committee, Victoria, Australia, Application Number 318/12 on the 23^rd^ October 2012.

### Theoretical Framework

A social constructionist approach was chosen as the framework for the study. From a social constructionist’s viewpoint, individual’s perceptions of reality and the meanings they give to phenomena are shaped by the social and cultural norms operating within that time and context [26]. The cultural and social meanings attributed to an illness can impact on the way in which an illness is viewed and experienced, particularly if an illness is stigmatized [27]. A condition is stigmatized not because there is something inherent in the condition itself but rather by the way society responds to the illness and how it manifests itself or the type of people who have the condition [28]. BV commonly manifests itself as vaginal malodor and abnormal discharge, symptoms or attributes which are commonly negatively perceived as associated with persons who are ‘unclean, sexually promiscuous or morally unsound’ [29] **.** The stigma of having a vaginal infection can greatly impact on women’s social and sexual lives as they feel considerable shame, anxiety, embarrassment and distress as a result of their vaginal symptoms [17], [21]. This in turn can influence the ways in which women cope with and understand their illness, and the ways they seek help and self-care [17], [21]. Given the symptoms of BV, we anticipated that women’s views and experiences of BV would likely be influenced by these broader negative social connotations.

### Method, Research Team and Reflexivity

Semi-structured interviews were chosen as the data gathering method as they allow the opportunity for women to tell their lived experiences and personal realities of recurrent BV while also allowing key areas of interest to clinical and research staff about recurrent BV, to be targeted. All interviews were conducted by JB or SW, both of whom are experienced female researchers accustomed to talking to participants about sexual health issues. JB is a research fellow with a doctorate (PhD) in public health and SW is an experienced psychologist with a doctorate in health psychology. JB and SW had no prior relationship with the participants. Both JB and SW have a good understanding of the epidemiology of BV and anticipated that the experiences of heterosexual women may differ from the experiences of WSW as BV is more common among WSW. Participants were informed that the research study was being undertaken in an effort to better understand women’s experiences of recurrent BV.

### Recruitment

Table One outlines the sampling framework and eligibility criteria for the study. Purposive sampling was undertaken in order to explore potential differences in the experiences and impact of recurrent BV among a diverse range of women [30]. Women were recruited from a number of locations including the Melbourne Sexual Health Centre (MSHC), the largest sexual health clinic in Victoria, Australia; via a longitudinal BV study of Australian women who have sex with women (WSW) [31] **;** and through medical clinics specialising in sexual health or general practices with a high case load of female patients of reproductive age (hereafter referred to as high caseload clinics). Women attending MSHC were opportunistically invited to take part in the study by a clinician or nurse during their consultation, women in the longitudinal BV study were invited to participate by letter, and study leaflets and posters were placed at four high caseload clinics across Melbourne inviting women to participate. Interested participants were referred to a study free call number where contact and eligibility details were collected. Women were then contacted shortly after to explain the study in detail, confirm eligibility and arrange an interview date and time. A total of three attempts were made to contact women before they were deemed lost to follow up.

**Table 1:** Sampling framework, eligibility criteria and interview schedule topics.

**Sampling framework**
• Heterosexual women and women who have sex with women (WSW)
• Single women and women in a relationship
• Recruitment locations - MSHC, high caseload clinics, longitudinal BV study
• High and low numbers of recurrent episodes of BV
***Revised during data collection to include***:
• Increased number of bisexual women (to compare the experiences of male and female sexual partners around BV)
• More women from high case load clinics (to compare and explore women’s experiences of a range of clinical settings in relation to BV*)
• More women with a higher number of episodes of recurrent BV (to compare experiences with women with fewer recurrences of BV)
**Eligibility criteria**
• Female
• Aged 18 to 45 years
• Two or more episodes of BV diagnosed by a clinician in <5 years
• Good understanding of written and verbal English
• Transgender participants must be born female, not had genital reassignment surgery or hormonal treatment (at the time of the interview).
**Interview schedule topics**
• First experience of BV
• Recurrent episodes of BV
• Impact on women’s social life, work life, sex life, relationships and emotionally
• Causes and triggers of BV *
• Experiences with the medical profession in relation to BV*
• Experience of treatment for BV*
• Support and information women would like available*
***Revised during data collection to include further questions about:***
• Levels of knowledge prior to having BV*
• How women felt when they had BV and why
• Partner’s fidelity as a result of having BV

* Study results for these topic areas are reported in an upcoming paper.

### Data Collection

Participants had the option of being interviewed either by telephone or face to face at MSHC or in their own home. All interviews were conducted between November 2012 and January 2013. Where possible, JB & SW were both present for interviews, with one note taking and the other interviewing. This technique was used as it allowed for consistency in interview style and technique, follow up questioning of the participant by the note taker at the end of the interview, and discussion, cross checking and interpretation of results, by the interviewers after the interview. Permission was always sought from women for both interviewers to be present with no women reporting that they were uncomfortable with this arrangement. No other persons were present at the time of interview.

Prior to the interview commencing participants were provided with a plain language statement (PLS) outlining the study and a consent form. Participants interviewed by telephone were read aloud the PLS and consent form, which was signed by the interviewer on their behalf. A copy of the PLS and consent form were provided to participants, in person or by post. All interviews were digitally recorded. Women were initially asked a series of 15 structured demographic, sexual behaviour and diagnosis and treatment questions which provided the interviewer with some understanding of their experience of BV prior to the semi-structured interview commencing. Table One outlines the topics women were asked about as part of their interview. Women were reimbursed with a $50 gift voucher for their time taking part in the study and were offered the option of checking and editing their interview manuscripts. Women who chose to receive their manuscripts were followed up two weeks after receiving them to check if they wanted any changes made. Only eight women elected to receive a copy of their manuscript for checking, and none requested changes.

### Data Pre-analysis and Analysis

JB and SW regularly met to discuss the results of the interviews and prepared a summary sheet detailing the participant’s experience and the interviewer’s impressions post interview about the impact of BV on women. Meetings were also held with the wider research team throughout the data collection stage to review interview transcripts, discuss preliminary results and further revise and refine the interview schedule and sampling framework. After approximately one third of interviews had been completed, the sampling framework and the interview schedule were revised (see Table One). When JB and SW felt that interview content was approaching saturation, the research team again met to review interview transcripts and discuss results, at which time it was decided by the group that no further interviews were required.

All interviews were transcribed and de-identified to protect confidentiality. Thematic analysis [32] was undertaken whereby each transcript was read by JB who began by manually coding responses, allocating a number to each code, grouping and labelling each code into broader themes and sub-themes and developing a key code. Data was coded using primarily a segmented approach [33]. Transcripts were then imported into N-Vivo 9 for data management, with the major themes and subthemes listed from the manual key code. Using the manual coding on transcripts as a guide, each transcript was again read by JB who further revised and refined the original coding, themes and subthemes in N-Vivo. Participant responses under each theme and subtheme were again further reviewed, refined and compared to determine similarities and differences. A subset of transcripts was reviewed independently by SW to cross check coding and themes. All coding and themes were confirmed by SW. Furthermore, a sample of transcripts were also separately reviewed by research team members (MTS, SW and JB) to examine any differences in women’s experiences in relation to their sexual identity (heterosexual and WSW), their relationship status (single women and women in a relationship) and engagement in sex industry work (sex industry workers and non sex industry workers). Each researcher reviewed twelve scripts from each group (i.e. six heterosexual and six WSW women). Analyses of demographic, sexual behaviour and diagnosis and treatment data were conducted using SPSS 20.0.

## Results

Of the 40 women who were referred to or registered their interest in the study, 35 completed an interview, 3 were ineligible after eligibility was checked again prior to organising an interview and 2 could not be contacted. Table Two outlines recruitment site details and participant characteristics. Interviews took between 20 to 45 minutes to complete depending on the depth of responses from participants. Twelve interviews were conducted at MSHC and 23 by telephone.

**Table 2:** Recruitment site and participant characteristics (demographic, sexual behaviour, diagnosis and symptoms of BV) N = 35.

	N or Median [Range]
**Recruitment site**	
MSHC	22
Longitudinal BV study	7
High caseload clinic	6
**Age**	30 [21–43]
**Born in Australia**	21
**Education level**	
Secondary school	5
TAFE diploma or certificate	9
Undergraduate degree	14
Post graduate certificate or degree	7
**Employment status**	
Full time	11
Part time	7
Casual	2
Student/Student & part time work	11
Unemployed	4
**Sexual Identity** #	
Heterosexual	19
Lesbian	7
Queer	3
Bisexual	4
Other (pansexual/transgender)	2
**Sex industry worker** ˆ	
No	29
Yes	6
**Smoke cigarettes**	
No	23
Yes	10
Past smoker	2
**Regular relationship**	
No	14
Yes	21
**Sex of partner**	
Male	13
Female	8
**Number of male sexual partners <5 years (if > = 1)**	10 [1–1300]
**Number of female sexual partners <5 years (if > = 1)**	3 [1–45]
**Number of times had BV in the past**	4 [2]–[35]
**Number of times had BV diagnosed in the past**	3 [2]–[25]
**Symptoms** *	
Abnormal odour	34
Abnormal discharge	35
**Most distressing symptom**	
Abnormal odour	30
Abnormal discharge	7

# Sexual identity refers to the label women were most comfortable using to describe their sexuality.

ˆ Includes sex workers, pornography model and dominatrix.

* Participants could choose more than one option.

### Episodes of BV

Women’s experiences of recurrent BV varied, with women experiencing anywhere from 2 to 35 recurrent episodes in the past. All women experienced BV-related symptomatology during an episode of BV - all 35 reported abnormal discharge and all but one reported malodour. Other genital symptoms women reported around the time of an episode of BV included itchiness, irritation, bleeding, pain during sexual intercourse, abdominal cramps and peeling skin around the vulva.

The following results present the overall impact of BV on women before detailing the physical, emotional, sexual and social impacts and the differences in experiences between groups of women.

### Overall Impact of BV

For just under a third of women, recurrent BV did not impact on their lives greatly, with some women equating it to being no more inconvenient than thrush.

*That’s it, it’s not like it so drastically affects your life that in fact it’s not really any worse than getting thrush. In fact it’s less bad than getting thrush (Participant 2, age 39).*

A number of these women considered having BV to be ‘just one of those things’, something that was annoying but not alarming or a little inconvenient.

*…it’s just a normal thing that, you know, a bit of an imbalance and you can correct it, and it’s not a big deal (Participant 13, age 22).*

While overall, women in this group did not seem overly concerned about having recurrent BV, many did still report feelings of embarrassment and self-consciousness around symptoms of BV, some impact on their sexual lives and concerns around possible long term sequelae associated with BV.

However, for the majority of women recurrent BV impacted either moderately or quite severely on their physical, emotional, sexual and social lives, the degree of which was commonly associated with the frequency of recurrences and severity of their symptoms.

*[I feel] uncomfortable, not happy, um, I can, I’m always smelling something and always trying to control that and it’s on my mind constantly when I have it, all you think about … (Participant 29, age 26).*

*…I can smell the smell so strongly and I am so disgusted with how it smells, um, that I would prefer not to be, um, around other people. Cos to me it’s, it’s awkward and it’s embarrassing and I think I smell like a dead thing and it’s just a really disgusting, horrible position to be in (Participant 5, age 38).*

Table Three provides three case examples of the differing impacts of BV on women. The impact of recurrent BV tended to be more severe among women who had experienced a higher number of episodes (4+) and more severe symptoms of odour and/or discharge. Age did not tend to influence the degree of impact, however a couple of women felt they had been more distressed at having BV when they were younger.

…*when I had it when I was younger, it definitely felt more symptomatic, um, and it was generally more distressing (Participant 22, age 28).*

**Table 3:** Examples of low to severe impact on women with recurrent bacterial vaginosis – case scenarios of participants.

Low impact	Moderate impact	Severe impact
Participant 14 was a 40 year old WSW who had been diagnosed with BV four times in the past. Her first episode occurred approximately 10 years ago, the second approximately 5 years ago, the third a couple of years ago, and the fourth about a year ago. She has not sought treatment for the fourth episode. She does not feel that BV has impacted on her life much at all and doesn’t seem overly concerned or perturbed by her current symptoms. She reported that both current and previous sexual partners have had similar symptoms and questioned whether BV may have been transmitted by a previous sexual partner. She feels that triggers for her BV could be increased frequency of sex, insertive sex and the use of lubricant. She uses an over the counter medication aimed at balancing vaginal flora when she knows she is going to have an increased amount of sex as this seems to prevent the exacerbation of her BV. She is currently considering alternative treatments as advised by her naturopath and acupuncturist. She experiences increased discharge and odour at varying times of her menstrual cycle.	Participant 30 was a 31 year old heterosexual female who experienced BV three times in the past. At the time of her first episode she was a ‘working girl’ and unable to have sex for six months until her symptoms subsided - in particular pain while having sex. She now works as a dominatrix and has learnt more about BV as the women she works with have also had BV. When she has BV she feels frustrated, embarrassed and dirty. She attributes her BV to oral sex, hygiene practices and frequent washing. BV impacts on her work, social and personal sex life. When she has BV she will not let her boyfriend perform oral sex on her. She reported that the malodour is noticeable sometimes with movement which she is self-conscious about. The worst thing about having BV for her is not knowing the cause of it and concerns about the long term effects of recurrent BV on her fertility. She has tried various self-help remedies in an attempt to treat symptoms and prevent further recurrences of BV.	Participant 34 was a 42 year old transgender (female to male) man.* At the time of the interview he was pre-operative and not taking hormone medication. He has had BV at least 35 times since first being diagnosed with it in 2004, and been diagnosed with it 25 times by clinicians. He has only had female sexual partners in the past five years. When he first got BV he was with a female partner who had BV. He sought treatment for it however it recurred very shortly afterwards (his partner was not treated at the time). He experienced his third episode a few months later and since this time reports that every time he has sex he gets BV, generally after the first few times he sleeps with a new partner. It has impacted on his self-esteem and confidence as he feels he can no longer have sex without getting BV. He has also had partners report they can smell his malodour. He refrains from sex altogether during an episode of BV and will visit his doctor if he knows he is going to have sex so that he will have antibiotic treatment on hand when he gets BV after intercourse. He describes having BV as the ‘bane of his existence’ and wishes there was more known about its cause and preventative options. He feels incredibly frustrated that he cannot have sex as frequently as he wants because of BV.

* Transgender men generally prefer to use the male pronoun to describe themselves. In this case, he still has his vagina, and is having receptive penetrative sex with his female partner.

### Physical Impact of BV

The most distressing symptom for almost all women, regardless of the overall impact, was unquestionably the malodour, however many women also found the abnormal and often profuse discharge very distressing. Table Four provides quotes of women’s experiences and feelings about their symptoms of BV. For most women the malodour was always present when they had BV however, for some the odour was only noticeable or worse during or after sex.

*…it was always worse after sex as well, the smell was always worse, and so, it’s not just the kind of dealing with the odour in the moment, but there’s afterwards and I think, you know, the semen pH changes everything and, and, um, even if he didn’t ejaculate inside me there was still [a] really strong, noticeable smell afterwards which is another disincentive (Participant 7, age 39).*

**Table 4:** Examples of women’s quotes on the physical symptoms of BV.

Malodour	Discharge
*It was a horrible fishy smell (Participant 12, age 30)*	*… the discharge that I had before, where, like, at, at some point I had it running down my leg, this is how, um, like heavy the discharge was. (Participant 16, age 27)*
*…then it deteriorated into this really foul smell where I was* *not comfortable (Participant 16, age 27)*	*…it was just a very horrid discharge and smelt and it just felt unusual (Participant 24, age 22)*
*…it was like a faint smell of, ah, like garbage almost of. Yeah, it was* *just a foul odour. It, it was, I think, fishy (Participant 17, age 34)*	*Yeah, well, yellowy creamy. I’ve never had the green stuff again… I get a discharge, it’s more smelly…and then that kind of lumpy, lumpiness…like, um, porridge or something (laughter) (Participant 19, age 34*
*I would say it smells like dead fish (laughs) it is. Yes it’s very* *uncomfortable because the amount of discharge is just too much* *(Participant 35, age 30)*	*Sometimes you forget in your daily activities and then when you relax, like sit down and do what you want then when you got to the bathroom and you see, yeah, you see dirty [discharge], like it has changed from morning to afternoon, and it’s just the, just the discharge, it’s just very disappointing…(Participant 8, age 28)*
*It’s just very different from a natural, normal healthy smelling vagina.* *It’s um, to be blunt, it is probably quite, um, piquant sort of strong* *scent. And you notice it on yourself a lot (Participant 3, age 39*)	*And discharge like I have to change my clothes all the time just because I don’t like the feeling of having to go to the toilet and taking my underwear off and like having this left on there or something like this (Participant 33, age 28)*
*…it’s a particular kind of smell that I would recognise (laughs) that I haven’t* *experienced since then (Participant 2, age 39)*	*…the discharge was another, it was completely different, and it was kind of ‘Hang on, what’s going on?’ (Participant 6, age 31).*
*…just a really musky smell. Like a really distinctive sort of… yeah, yeah.* *Yeah like you could wash and then like an hour later… you could wash* *all day (Participant 23, age 24)*	
*…the first time that I got it, I thought there was something seriously* *and desperately wrong with me because the smell is atrocious…Yeah and the only* *thing I can liken it to is a dead cat (Participant 5, age 38).*	

While most women strongly disliked the malodour, one woman reported she actually quite liked the smell and did not find it offensive -‘*…and then that smell, which, you know, I like*’ (Participant 19, age 34). This woman had experienced BV three times in the past and found that she was able to effectively treat her symptoms - which were not severe - through douching with vinegar and water. BV had had a very limited impact on her work (as a sex industry worker), social or personal sex life.

### Emotional Impact of BV

For most women having BV made them feel embarrassed, self-conscious and uncomfortable, with many women also reporting they felt disgusted, ashamed, dirty, annoyed and distressed. Table Five provides quotes on the different feelings women experienced around having BV.

**Table 5:** Example of women’s quotes on the emotional impact of BV.

How BV makes women feel	
*Dirty*	*I feel unclean. I wanna shower all the time because I just feel, I don’t know, I just feel dirty (Participant 25, age 37)*
	*…Ok, it makes me feel dirty. It makes me feel you know, not really that keen to have sexual… you know relationships with me. Um, it’s really embarrassing, and… I just really don’t like it. It’s just really yeah horrible (Participant 15, age 37)*
*Embarrassed and ashamed*	*…I had a new lover and I was travelling and it came back and I was so embarrassed and ashamed about it (Participant 33, age 28)*
	*Well I don’t because like I said it’s, well… embarrassing because I don’t always know when it’s starting and yeah, so… it can be quite embarrassing (Participant 34, age 42)*
*Annoyed*	*[It makes me feel] annoyed because it’s just sort of, annoyed because it restricts, like I’m dating and it restricts, ah, what I’m about to do with different people (Participant 20, age 23)*
*Self-conscious*	*It does make you feel self-conscious, definitely (Participant 3, age 39)*
*Uncomfortable*	*…it does feel disgusting because, just, my clothes smell, and it just, mmm, not comfortable (Participant 16, age 27)*
	*Very uncomfortable as in, cos of the smell and discharge make me very alert of, I don’t know, I’m just feeling very uncomfortable being around people (Participant 35, age 30)*
*In poor health or unwell*	*…anything that involves discharge and odour you are really aware of the fact that you are sick in some way (Participant 2, age 39)*
	*…my moods are different, yes I’m not as happy, I’m uncomfortable and have something wrong with me (Participant 29, age 26)*
*Emotionally distressed*	*…Very, very important just how you feel, even though it’s physically happening to you, mentally you are thinking it over and over, making it worse sometimes (Participant 8, age 28).*
	*…I mean it’s not debilitating in terms of physically debilitating, but its debilitating, you know, mentally and emotionally (Participant 15, age 37)*

#### Lack of control

For the majority of women one of the most difficult aspects of having recurrent BV was the confusion, frustration, helplessness and disappointment they felt about why they were experiencing BV and their lack of control over recurrence.

*So just really confused so everything was running through my head, is it the Mirena coil? Has my boyfriend cheated on me and given me something? Have I done something wrong?*

*Have I lost a tampon up there?…Did I change my soap? Like confused. I was just like ‘What have I done to do this?’ (Participant 26, age 24)*

*…I don’t feel like I know what it is, what is the behaviour or the action to know what is causing it so I don’t know why I’m getting it (Participant 20, age 23)*

Women commonly reported always worrying about having BV, feeling relieved when they did not have an episode, wishing recurrences would stop, but fearing or thinking they inevitably never would.

*Very frustrated and I was just like, ‘Oh my God, do I have to live with this for the rest of my life!?’ and I just thought that it’s ridiculous. Yeah I was really frustrated (Participant 15, age 37).*

*I already accepted the fact that I have BV. I already kind of accepted that I’m not going to get rid of it (Participant 33, age 28)*

#### Long term sequelae

A number of women also raised concerns around the possible long term sequelae associated with recurrent BV, mainly reproductive sequelae.

*… I guess at this current moment the stress to me, or the, um, the difficulty is uncertainty as to how BV might relate to fertility issues and childbirth issues (Participant 22, age 28).*

*…I saw on that* Embarrassing Illnesses *show [British television series] about bacterial vaginosis and it said that it effects fertility especially if it keeps coming back (Participant 30, age 31).*

#### Shame and stigma around BV

For many women, their feelings of embarrassment, shame and frustration at having BV adversely impacted on their self-esteem and confidence, leaving them feeling unattractive and insecure.

*…obviously give[s] you very less confidence, less confident, you feel insecure, even though you [are] not having sexual intercourse you don’t want to be very close to people cos you can smell it, you don’t want people to smell it (Participant 8, age 28).*

A number of women felt that the shame and embarrassment they experienced around BV could be attributed to broader societal stigma attached to woman’s sexuality, sexual functioning, promiscuity and STIs.

*…it’s quite isolating, because women don’t talk about it, and there must be, must be women in my life who, who have had it, and, and I mean we just don’t talk about it, it’s that shame I suppose, embarrassment, and…There’s, I mean, there’s, we’ve got culturally all this stuff about vaginas anyway, and, um, and the fishy thing, it’s not normal. And so I think, you know, it the one thing to talk about our periods or our menstrual cycle or even talking about sex I think is easier than talking about BV. And that’s not easy. I think, there must be shame, it must be… (Participant 7, age 39).*

*Like I think people are really, I think women are very sensitive about vaginas and talking about them and, sort of, even thinking about their own, you know, body, because it’s such a taboo topic in, you know, in mainstream society you, guys talk about their penises all the time, and girls would, like we’d never sit down and like say like ‘How’s your vagina going today?’ You know what I mean? (Participant 13, age 22).*

Interestingly, a number of women equated sexual attractiveness with a non-odorous vagina.

*…it’s an unpleasant smell that’s sort of, it’s the kind of thing people joke about, like having a bad smelling vagina (laughs) is not really an attractive attribute (laughs). It’s like, ‘Oh yeah, I’m that woman’ (laughs) (Participant 1, age 25).*

*…the very strange odour that’s, um, not really associated with attractive smelling girls… (Participant 10, age 23).*

It was also very common for women to associate being ‘clean’ and having good hygiene practices with a lack of BV, and yet, at the same time to feel confused as they were experiencing recurrent episodes in spite of maintaining good hygiene practices.

*… we have to be clean, um, and so we do stay very clean. So it’s very frustrating, um, yeah to keep getting it recur (Participant 5, age 38).*

*… I just don’t feel like I should be having it, you know? Because I am pretty hygienic and I don’t have all these things and yeah, so I don’t have any clue why it’s causing it (Participant 8, age 28).*

### Impact on Sexual Relationships

The biggest impact of BV was unquestionably on women’s sex lives and sexual intimacy, due to their strong concerns that sexual partners would notice ‘the smell’. Women’s embarrassment and fear of sexual partners noticing their symptoms meant they often avoided certain sexual practices, particularly oral sex, abstained from sex altogether, and avoided certain sexual positions.

*I think it has in terms of like I have not slept with people because of that smell or like avoiding guys going down on me at times cos I’ve been like yeah that’s embarrassing (Participant 1, age 25).*

*…like I couldn’t be intimate with my partner, so it was really upsetting… it’s not really having sexual intercourse with my partner, it’s not that. It’s just being next to someone that, that you’re cuddling or whatever, and if I smell it, and having to experience that is not pleasant (Participant 16, age 27).*

Women who continued to have sexual contact with their partners, generally felt very embarrassed, self-conscious and unable to relax and enjoy sex.

*I was so embarrassed and ashamed about it…she said she doesn’t mind and had sex with me even though I have this very strong smell and this was very hard for me and immediately when we had finished I would take her to the bathroom and make her wash her hands and make sure the smell was gone. And even then I would feel like the smell was still there and it makes me not want to have sex anymore (Participant 33, age 28).*

*… So mentally it’s a thing for me because if you can’t completely let yourself go and be in bed with your partner then that’s not really good is it? (Participant 32, age 43).*

Only a few women reported that their sexual practices had not really changed greatly or at all as a result of having BV.

*I sort of would avoid it at first, but then I like oh well, let’s do it anyway, and so I didn’t really avoid it (Participant 13, age 22).*

BV not only affected women’s sex lives and practices but also their sexual self-esteem, sexual confidence and levels of intimacy and closeness with their partners.

*… I guess once it happens I don’t want to have sex, um, it also affects my self-esteem. Or well my sexual confidence I guess, I dunno, I guess I kind of, um… I don’t know how to explain it, I guess it just puts me off wanting to be intimate (Participant 34, age 42)*

One participant in particular was extremely distressed about the impact recurrent BV was having on her sex life and relationship with her partner.

*…(sobbing) just the smell like, um, yeah it’s the worst like it’s… it makes things, um, like my boyfriend’s great about it, but the worst thing is like I don’t wanna have sex cos I don’t want it to be unpleasant kinda thing and he does and it upsets him because he wants me to enjoy it as much as him kinda thing (sobbing) like, um, like he says he wants to go down on me and all that sort of stuff but I don’t want him to and I dunno I guess it’s like that’s shit. And then like a few weeks ago, um, he said it was like starting to get to him kinda thing and, um, like it’s fine, it’s not gonna end things or make things bad but it’s just kinda, it’s annoying… (Participant 27, age 21).*

Interestingly, while women’s greatest fear was that their sexual partners would notice their symptoms, most partners were either unaware of women’s symptoms or unconcerned, showing high levels of support and understanding.

*I was embarrassed for my boyfriend to go down on me, even though when he did he, he’d said he didn’t smell anything (Participant 17, age 34).*

Most women were able to talk openly with their partners about BV, more commonly with regular rather than casual partners, and while many partners did not really understand what BV was, most did not want women feeling uncomfortable or inhibited because of it.

*I felt really able to talk, talk about it with my partner. It wasn’t something that we couldn’t really discuss…And he was very reassuring and encouraging and wanted me to find out what was going on and to not put up with it. He has been very supportive all along (Participant 7, age 39).*

*You know, so, we make jokes about it. She says BV says a bad vagina (laughs), so, you know, it’s been a source of humour (Participant 19, age 34).*

A few women however did not discuss their diagnosis or symptoms of BV with partners as they were too embarrassed or did not think their partners would understand what BV was.

*I just try and hide it and, um, you know just make sure that I’m showered and you know how embarrassing! (laughing) (Participant 15, age 37).*

*…with partners it’s a little more difficult because I don’t think they understand that it’s an overgrowth of your own natural bacteria caused by sex, it’s a hard concept because then they think if it’s caused by sex it must have been a germ passed on that I’ve caught (Participant 24, age 22).*

One participant did not tell her partners because it was not an STI and therefore did not feel she had an obligation to inform them.

*…because it’s not really, and from my understanding it’s not really an STI so it’s not like if I have sex I feel that I need to, um, tell a partner and like I said none of my past partners have ever gotten it other than the most recent one…(Participant 34, age 42).*

For a few women that did tell their partners however, their fears and embarrassment were reinforced when partners reacted negatively to their symptoms or avoided sexual contact.

*The worst part was when my boyfriend noticed and was like, ‘What is that?’ and stopped having sex with me and I got really embarrassed (Participant 26, age 24).*

*I didn’t use condoms before but when I had these symptoms I started to use a condom and he [partner] didn’t like it and yeah like I guess having sex and it affect our relationship and I was uncomfortable with it. I think that it affected our relationship to get worse. I explained to him but he thought it was about, not a big problem and, um, so he didn’t wanna use a condom but, ah, I wanted to use a condom because it’s like a disease and I don’t want him to get something. But he didn’t care about that and our relationship get worse, yeah. (Participant 9, age 28).*

Interestingly, women did not report questioning their partner’s fidelity or vice versa as a result of having BV, although one woman had previously been alerted to her partner’s infidelity when she was also diagnosed with chlamydia during a routine medical check-up.

### Social Impact – Work and Social Life

Women’s concern that others would detect their abnormal odour also impacted on their social and work lives. Some women reported avoiding social situations, recreational activities, standing or sitting too close to others, or even borrow friend’s clothes for fear that others would notice the odour.

*…so when I go to school when I’m sitting in, ah, a room full of people, um, and especially in lecture halls where the, um, the chairs are very, very, close together, um, having a problem like BV… um, it’s a problem because I know I can smell it even when I have my legs firmly closed or when I cross my legs or… I can smell it, I know that it’s, I’m very aware of the fact that it’s there so if I change my legs from being crossed one way, changing them and crossing the other, I can smell the smell so strongly… (Participant 5, age 38).*

*Well definitely I did keep a distance away from my friends. I didn’t want them to sit very close to me. Yeah the smell it’s, is embarrassing. Yeah I still go out but I make sure that I wash myself before I go out and really keep an eye on it and don’t stay out too long (Participant 35, age 30).*

Most women reported that having BV did not impact on their work. Only a few women reported feeling self-conscious or uncomfortable at work when they had BV. Women working in occupations that required close contact with people such as teachers, physiotherapists, healthcare workers (i.e. nurses, dental assistants, physiotherapists) or sex industry workers were more likely to be impacted.

*…because I’m a [healthcare worker] I have quite close contact with people and I sometimes get worried that, well I have to make sure that I’m nice and clean so that the odour doesn’t, um, you know, so that my patients don’t notice the odour. So that can be a bit embarrassing or worrying for me sometimes… (Participant 14, age 40).*

*…I’m a [healthcare worker] so working in a […] clinic and especially when a patient’s head is right near your lap as well that was awful (Participant 4, age 40).*

Unlike non-sex industry workers, the majority of sex industry workers reported that they were either unable to, limited in, or did not want to work when they had an episode of BV, which affected their earning capacity and financial security.

*I guess the stress is because it does affect my work and therefore my ability to… you know to support myself financially (Participant 22, age 28).*

*I work in the sex industry, um, I don’t work at all when I’ve got BV, I do not work at all cos… I can’t, I won’t… it’s unacceptable to be in that condition, um, and yeah so I kind of take myself away until, um, I have the opportunity to get the medication and, um, be clean. (Participant 5, age 38).*

Of the six sex industry workers, only two reported that BV had little impact on their work or life in general, however one woman had only experienced one of her three episodes while working.

### Differences between Groups of Women

As part of the study we explored possible differences in the experiences of a number of groups of women including: heterosexual women and WSW, single women and women in a relationship, and sex industry workers and non-sex industry workers. While we anticipated that there may be differences, in particular among heterosexual women and WSW, no consistent differences were observed and bisexually active women reported no notable differences in male and female partners’ responses or levels of support around BV compared to heterosexual women or WSW. Single women were less likely to discuss their BV diagnosis with casual partners and more likely to discuss it with friends. Women in relationships were more likely to discuss their concerns with regular partners, but also commonly discussed their diagnosis with friends as a means of information gathering. Women in relationships tended to experience greater support as they had both partners and friends to discuss their diagnosis with. The major difference observed between groups were among sex industry workers and non-sex industry workers where sex industry workers experienced a greater impact on their work life than non-sex industry workers.

## Discussion

Women’s experiences of recurrent BV and its effect on their lives varied. While some women reported BV did not impact greatly on their lives, for the majority, it moderately to severely impacted on their lives, causing them a great deal of distress and anguish. Almost all women experienced symptoms of malodour and abnormal discharge, and as has been commonly reported, symptoms were worse during or following sex for some women [4], [5]. The degree of impact was commonly related to the frequency of episodes and the severity of symptoms. Women with a higher number of recurrences and more severe symptoms tended to report a greater impact. These study findings expand existing knowledge on women’s experiences of recurrent BV. Unlike in previous research [21], women in this study did not appear to question their partner’s fidelity as a result of having a vaginal infection. They did however have concerns about their reproductive morbidity [21]. Most women felt considerable embarrassment and shame around their symptoms of BV, some who attributed this to broader societal stigma around women’s sexuality, behaviour and STI’s. Many women were confused about why they were experiencing recurrent BV and, frustrated about their lack of control over recurrences.

### Strengths & Limitations

A limitation of this study was that women were not required to show evidence of previous diagnosis of BV by a medical practitioner, therefore we cannot be certain that all women had been professionally diagnosed with BV two or more times in the past five years; it is possible that some women’s recollections of diagnosis could have been inaccurate. The majority of women however, reported experiencing three or more episodes of BV in the past even if they had only been professionally diagnosed twice.

The strength of this study is that it not only adds to the very limited data available about women’s experiences and the impact of recurrent BV, but it also examines a diverse sample of women. One previous qualitative study has explored the impact of recurrent BV and its treatment on African American women’s quality of life and lifestyle practices [17], whilst a further study has examined the experiences of women with BV as part of a broader study of women with vaginitis (thrush, BV and trichomoniasis) in a primary care setting [21]. Our study focused specifically on BV and included heterosexual women and WSW - including a transgender participant - women from a range of ethnic backgrounds, ages and socioeconomic status and sex workers, who have their own unique and highly informed perspective on BV.

In offering a combination of both face-to-face interviews and telephone interviews we were able to include a wider variety of participants and found more often than not, that women interviewed by telephone were freer in disclosing personal information than women interviewed face-to-face. This generally resulted in equally, if not richer data, than that collected in face-to-face interviews. This finding is not uncommon [34] with previous studies showing no significant difference in the quality of data collected between telephone and face-to-face interviews [35]. It is likely that for many women, discussing issues of such a personal nature was easier to do over the telephone. The research team discussed this finding at meetings however no formal assessment was undertaken to evaluate whether mode of interview had an impact on interview content.

Our study suggests that for many women recurrent BV is a distressing condition that can have a major impact on their self-esteem, sexual relationships and quality of life. These findings are supported by previous studies which have also found that women with vaginal conditions, in particular chronic or recurring conditions, experience considerable distress, anxiety and frustration, which can severely affect their quality of life to a far greater extent than currently acknowledged. In the aforementioned study of African American women experiencing recurrent BV, women commonly reported feelings of shame, embarrassment and frustration at having recurrent BV [17]. They also recounted social and sexual avoidance behaviours, including avoiding others at work and in social situations, abstaining from work and social events altogether and avoiding or abstaining from sexual activity due to self-consciousness around vaginal malodour [17]. In the broader qualitative study of women’s experiences with vaginitis [21] researchers also found that vaginal symptoms caused women great anxiety and distress, which could severely impact on their social and sexual lives. Women reported concerns around their long term reproductive health and were more likely to feel that symptoms were indicative of something more serious if they were chronic rather than transitory [21]. Other studies examining the experiences of women with thrush have also found that vaginal symptoms can impact majorly on women’s social, emotional and sexual lives [36], [37].

Our study provides greater depth than previous studies regarding the experiences related to sexual partners. While a few women concealed their BV from partners, most were open about it, received support and encouragement from their partners and did not report any concerns around sexual infidelity. These findings differ from those found by Karasz and Anderson [21] who found that women either did not disclose or selectively disclosed their vaginal symptoms - most often to family or friends – for fear others may assume they were sexually promiscuous or that it may trigger arguments with their partners around infidelity – either theirs or their partners [21].

Unfortunately, disclosure to partners and the psychosocial impacts of vaginal conditions such as BV are not well acknowledged or reported in the research literature. BV, like many vaginal conditions, is often regarded as a non-serious, genital condition and commonly described by practitioners as an imbalance of vaginal flora that can be treated with antibiotics [4], [5]. Whether BV is sexually transmitted remains unclear and somewhat controversial. While it is commonly acknowledged that considerable psychosocial sequelae and social stigma are associated with STI diagnoses [22]–[24], [29], [38] there have been very limited attempts to investigate whether women with vaginal conditions such as BV also experience similar feelings of shame, stigma and psychosocial sequelae. Given that, symptoms of vaginal malodour are commonly associated with unattractiveness, poor hygiene and/or sexual promiscuity, symptoms may be perceptible by others, BV is recurrent in nature, and there is considerable confusion on the part of both clinicians and patients as to whether BV is sexually transmitted, it is not surprising that women are experiencing poor self-esteem, sexual withdrawal, self-isolation and feelings of self-blame.

### Future Implications

When managing women with recurrent BV it is important to recognise not only the physical symptoms or discomfort of BV but the significant and distressing psychosocial sequelae experienced by women. While BV is often considered a minor and common vaginal condition by clinicians, its recurrent nature and the substantial impact it can have on women’s emotional, sexual and social lives means that women’s experiences can extend far beyond the physical symptoms. Recognition of psychosocial sequelae should be applied to any recurring vaginal condition that may be socially stigmatised and have symptoms which can cause great distress to women, impacting heavily on their quality of life.

Further studies are required to confirm the extent of psychosocial sequelae experienced among varied populations of women with recurrent BV and determine the support and information women and clinician’s require for the effective management of recurrent BV.
